# A Whole Genome Sequencing-Based Genome-Wide Association Study Reveals the Potential Associations of Teat Number in Qingping Pigs

**DOI:** 10.3390/ani12091057

**Published:** 2022-04-20

**Authors:** Zezhang Liu, Hong Li, Zhuxia Zhong, Siwen Jiang

**Affiliations:** 1 Agricultural Ministry Key Laboratory of Swine Breeding and Genetics & Key Laboratory of Agricultural Animal Genetics, Breeding and Reproduction of Ministry of Education, Huazhong Agricultural University, Wuhan 430070, China; zzl19920507@hotmail.com (Z.L.); zzx05031319@163.com (Z.Z.); 2 Novogene Bioinformatics Institute, Beijing 100083, China; lihong@novogene.com

**Keywords:** teat number, Qingping pig, whole genome resequencing, *TBX3*, Wnt signaling pathway

## Abstract

**Simple Summary:**

Teat number is important for the mothering ability of a sow, but this trait has seldom been investigated by high-depth genomic data-based genome-wide association study (GWAS). Here, we performed GWAS for the teat number-related traits in 100 Chinese native Qingping pigs by recording their left and right teat numbers and analyzing their genetic variations through 10-fold whole-genome sequencing. *T-Box Transcription Factor 3* (*TBX3*) on *Sus scrofa* chromosome (SSC) 14 and Wnt signaling pathway are revealed to be associated with teat number-related traits, with important roles in mammary gland morphogenesis and development.

**Abstract:**

Teat number plays an important role in the reproductive performance of sows and the growth of piglets. However, the quantitative trait loci (QTLs) and candidate genes for the teat number-related traits in Qingping pigs remain unknown. In this study, we performed GWAS based on whole-genome single-nucleotide polymorphisms (SNPs) and insertions/deletions (Indels) for the total number of teats and five other related traits in 100 Qingping pigs. SNPs and Indels of all 100 pigs were genotyped using 10× whole genome resequencing. GWAS using General Linear Models (GLM) detected a total of 28 SNPs and 45 Indels as peak markers for these six traits. We also performed GWAS for the absolute difference between left and right teat number (ADIFF) using Fixed and random model Circulating Probability Unification (FarmCPU). The most strongly associated SNP and Indel with a distance of 562,788 bp were significantly associated with ADIFF in both GLM and FarmCPU models. In the 1-Mb regions of the most strongly associated SNP and Indel, there were five annotated genes, including *TRIML1*, *TRIML2*, *ZFP42*, *FAT1* and *MTNR1A*. We also highlighted *TBX3* as an interesting candidate gene for SSC14. Enrichment analysis of candidate genes suggested the Wnt signaling pathway may contribute to teat number-related traits. This study expanded significant marker-trait associations for teat number and provided useful molecular markers and candidate genes for teat number improvement in the breeding of sows.

## 1. Introduction

Porcine teats are located from the anterior to posterior limb bud and symmetrical to the abdominal midline, and teat number is an important trait for the mothering ability of a sow, which can affect the piglets’ weight gain and mortality. It has been shown that teat number is a quantitative trait with a medium level of heritability (0.32) [1]. Using genetic markers can speed up the genetic improvement of teat number. Thus far, 655 teat number quantitative trait loci (QTLs) in pigs have been reported and included in the PigQTL database [2]. Previous genome-wide association studies (GWAS) found microsatellite markers or single nucleotide polymorphisms (SNPs) in popular breeds, including Durocs [1,3], Large Whites [4], and their crosses with Meishan pigs [5,6]. GWAS were also performed for teat number-related traits in several Chinese native pig breeds, including Erhualian [7], Sushan [8] and Beijing black [9]. However, the genetic architecture for teat number in Qingping pig, a Chinese native pig breed, is still not clear.

In 2000, Wada et al. revealed two QTLs for teat number on SSC1 and SSC7 by QTL analysis of 265 F2 offspring of the Meishan and Göttingen miniature pig [10]. Since then, an increasing number of studies have used microsatellite markers to confirm the QTL on SSC7 in other pig populations, including Meishan × Duroc F2 resource population [5], F2 populations of Yorkshire boars and Meishan sows [11], and Meishan × Large White F2 pigs [12]. Additionally, a number of studies detected associated SNPs for teat number near or within this QTL on SSC7 in Duroc pig [1,3,13,14], White Duroc × Erhualian F2 resource population [15], a commercial swine population [16], and Large White pig [17]. Notably, *VRTN* (located at 103.4 Mb on SSC7 on *Sscrofa*10.2) was a credible candidate gene in this major QTL for teat number on SSC7. These studies also indicated that QTLs for teat number-related traits are distributed across the genome on every chromosome. Several other genes have also been annotated as teat number-associated genes, such as Lysine Demethylase 6B (*KDM6B*) [15], TOX High Mobility Group Box Family Member 3 (*TOX3*) [17], Estrogen Receptor 1 (*ESR1*) and Nuclear Receptor Subfamily 5 Group A Member 1 (*NR5A1*) [8]. However, most previous studies have not used the high-density single-nucleotide polymorphisms (SNPs) detected by high-throughput sequencing data to investigate teat number-related traits.

The purpose of the current study was to detect genome-wide associations for teat number-related traits in Qingping pigs using whole-genome SNPs and insertions/deletions (Indels) based on whole-genome resequencing.

## 2. Materials and Methods

### 2.1. Sample and Sequencing

In this study, all 100 pigs were raised indoors in the Qingping pig Conservation Farm in Yichang, Hubei, China. Ear tissues were collected and stored in liquid nitrogen until further analysis. Genomic DNA samples were extracted from ear tissues, using a standard phenol–chloroform method. Sequencing libraries were constructed and sequenced by the Novogene Bioinformatics Institute (Novogene, Beijing, China). High-throughput sequencing was performed as paired-end 150 sequencing using a HiSeq 4000 sequencing system (Illumina, San Diego, CA, USA).

### 2.2. Phenotypic Data

In this study, three independent persons observed all 100 pigs and counted the teat number of the left and right lines. A total of six teat number-related traits were obtained: (i) the number of teats on the left side (LTN); (ii) the number of teats on the right side (RTN); (iii) the total number of teats (TNUM = LTN + RTN); (iv) the maximum number of teats in LTN and RTN (MAXAP); (v) the difference between the two sides (L-R = LTN − RTN) and (vi) the absolute difference between left and right teat number (ADIFF = |LTN − RTN|). The mean, standard deviation, minimum value, maximum value, and coefficient of variance for each trait were calculated using R (version 3.6.0) (R Core Team, Vienna, Austria).

### 2.3. Genotyping and Quality Control

Clean reads from all 100 pigs were aligned to the *Sscrofa11.1* reference genome by the BWA software (version: 0.7.8) (Wellcome Trust Sanger Institute, Hinxton, UK) [18]. To reduce mismatches generated by PCR amplification before sequencing, duplicated reads were removed using SAMtools (Wellcome Trust Sanger Institute, Hinxton, UK) [19]. SNPs and Indels calling were initially performed to generate a gvcf file using UnifiedGenotyper in GATK (version 3.6) (Broad Institute, Cambridge, MA, USA) [20]. SNPs and Indels were divided using SelectVariants in GATK. Hard filtering of SNPs was applied to the raw variant set using “QUAL < 30.0 || QD < 2.0 || FS > 60.0 || MQ < 40.0 || SOR > 3.0 || ReadPosRankSum < −8.0”. Hard filtering of Indels was applied to the raw variant set using “QUAL < 30.0 || QD < 2.0 || FS > 200.0 || SOR > 10.0 || ReadPosRankSum < −20.0 || MQ < 40.0 || MQRankSum < −12.5”. SNPs and Indels in the VCF files were quality-filtered using VCFtools (v0.1.16) (Wellcome Trust Sanger Institute, Hinxton, UK) to remove variants with sequencing depth less than 3 [21]. SNPs and Indels were further filtered using PLINK 2.0 (Complete Genomics, Mountain View, CA, USA) [22]. Missing genotypes were imputed using beagle.03Jul19.b33.jar [23], followed by filtering SNPs and Indels again using PLINK 2.0 to obtain the high-quality common SNPs and Indels of 100 pigs for further analysis (individual call rate > 0.90; minor allele frequency > 0.05; call rate > 0.90, SNPs and Indels in Hardy–Weinberg equilibrium (*p* > 1 × 10^−6^) and excluding SNPs and Indels located on the sex chromosomes).

### 2.4. SNP-Based Heritability

The phenotypic variance explained by genome-wide SNPs (SNP-based heritability) was estimated using GREML in Genome-wide Complex Trait Analysis (GCTA) [24]. Briefly, the genetic relationships between pairwise individuals from all the autosomal SNPs were estimated using the genetic relationship matrix (GRM) based on high-quality common SNPs, followed by GRM and phenotype for restricted maximum likelihood (REML) analysis to estimate the variance explained by the SNPs.

### 2.5. Principal Component Analysis

Principal component analysis (PCA) was used to explore the population structure of Qingping pigs and determine whether principal components (PCs) should be added to the GWAS. PCA was performed using MVP.Data.PC in rMVP R (version 3.6.0) (Huazhong Agricultural University, Wuhan, China) [25] package based on high quality common SNPs. PC1 and PC2 were visualized using MVP.PCAplot in rMVP R (version 3.6.0) package.

### 2.6. GWAS Using General Linear Model (GLM)

In the present study, GLM in rMVP R (version 3.6.0) package was used to perform the GWAS for teat number-related traits based on high-quality common SNPs and Indels [26]. Principal component analysis showed no discernible clustering. Therefore, no principal component was adjusted in the subsequent association analysis. Each SNP or Indel for teat number-related traits was tested by GLM as follows:

y=Xb+e

where y is the vector of each teat number-related trait in Qingping pigs; X, a matrix of test SNP or Indel; b, an incidence vector for X; e, a vector of residuals following a normal distribution with a mean of zero and $I\sigma_{e}^{2}$ covariance, where I is the identity matrix and $\sigma_{e}^{2}$ is the residual variance.

### 2.7. GWAS Using FarmCPU

The Fixed Effect Model (FEM) and the Random Effect Model (REM) are used iteratively in FarmCPU [27], and FarmCPU in rMVP R (version 3.6.0) package was also used to perform GWAS for ADIFF. In the GLM model, some genetic markers were significantly associated with ADIFF with a whole-genome significant *p*-value (2.16 × 10^−9^ for SNPs and 2.44 × 10^−8^ for Indels). These markers can be used to define kinship in the REM step of FarmCPU to avoid the model over-fitting problem in FEM. The FEM is used to test each genetic marker, one at a time. Pseudo QTNs are included as covariates to control false positives. Specifically, the FEM could be expressed by the following equation:

$$y_{i}=M_{i1}b_{1}+M_{i2}b_{2}+\cdots +M_{it}b_{t}+S_{ij}d_{j}+e_{i}$$

where $y_{i}$ is the phenotype of the ith individual; $M_{i1}$, $M_{i2}$, …, $M_{it}$, the genotypes of t. pseudo QTNs, initiated with no QTN; $b_{1}$, $b_{2}$, …, $b_{t}$, the corresponding effects of the pseudo QTNs; $S_{ij}$, the genotype of the ith individual and jth genetic marker; $d_{j}$, the corresponding effect of the jth genetic marker; $e_{i}$, the residual having a distribution with zero mean and variance of $\sigma_{e}^{2}$.

The REM is used to optimize the selection of pseudo QTNs from markers with whole genome significant *p*-values (2.16 × 10^−9^ for SNPs and 2.44 × 10^−8^ for Indels) and positions by using the SUPER algorithm [28]. The REM could be expressed by the following equation:

$$y_{i}=u_{i}+e_{i}$$

where $y_{i}$ and $e_{i}$. are the same as in FEM, and $u_{i}$ indicates the total additive genetic effect of the ith individual. The expectations of the individuals’ total genetic effects are zeros. The variance and covariance matrix of the individuals’ total genetic effects can be expressed by $G=2K\sigma_{a}^{2}$, where $\sigma_{a}^{2}$ is an unknown genetic variance and K is the kinship matrix calculated by pseudo QTNs. The FEM and REM are iterated until no new pseudo QTNs are added, or the specified maximum number of iterations is reached.

### 2.8. Comparison with Known QTLs and Haplotype Analysis

Pig QTLs based on *Sscrofa11.1* were downloaded from the PigQTL database. The information of QTLs for teat number-related traits was obtained using R (version 3.6.0), followed by comparing the significant SNPs and Indels with these QTLs using R (version 3.6.0). For the strongest significant SNP (rs322863105) for ADIFF on SSC17, SNPs with suggestive significant *p*-values (1/557,540, 1.79 × 10^−6^) around rs322863105 were used for haplotype block analysis to evaluate the linkage disequilibrium (LD) patterns of selected SNPs within this region using Haploview version 4.2 [29]. The effects of this SNP on ADIFF were plotted using ggplot2 R (version 3.6.0) package.

### 2.9. Annotation of Candidate Genes and Functional Enrichment Analysis

Candidate genes, including or close to the significant SNPs and Indels, were annotated using the biomaRt [30] R (version 3.6.0) package based on *Sscrofa11.1*. Candidate genes located in 1-Mb regions of significant SNPs and Indels were also annotated. Gene ontology (GO) and Kyoto encyclopedia of genes and genomes (KEGG) pathway enrichment analyses and visualizations were performed using the clusterProfiler R (version 3.6.0) package [31].

## 3. Results

### 3.1. Genotyping and Phenotypic Statistics

**Figure 1:**
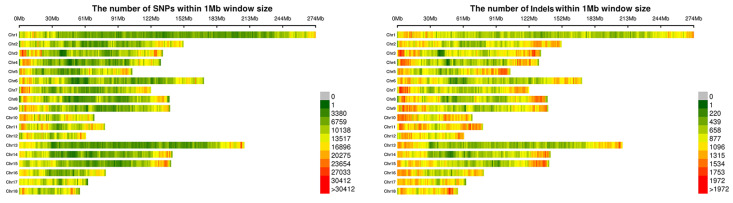
The distribution of SNPs and Indels on autosomal chromosomes of pigs.

After whole-genome sequencing, 2.76 TB of sequences were generated, with a mean coverage of 98.47% at an average of 9.66-fold depth for 100 Qingping pigs in this study (Table S1). Clean data were mapped to the pig reference genome (*Sscrofa*11.1), with 36,482,281 SNPs and 4,859,001 Indels being called with GATK. After filtering, 23,193,931 SNPs and 2,053,221 Indels, with a distribution roughly proportional to autosomal chromosomes of pigs, were retained for subsequent analyses (Figure 1).

Table 1 shows the descriptive statistics of teat number-related traits of Qingping pigs. The average teat number [standard deviation (SD)] was seen to be 14.78 (0.97), ranging from 14 to 17, which was higher than the values of Beijing Black Pig (13.6) [9], Japanese Duroc (13.73) [3], American (10.90) and Canadian Duroc (10.92) [14], but lower than the value of Erhualian pigs (19.13) [7]. The meansvalues (SD) for the other teat number-related traits, were 7.36 (0.48), 7.42 (0.61), 7.50 (0.59), −0.06 (0.51) and 0.22 (0.46) for LTN, RTN, MAXAP, L-R and ADIFF, with coefficient of variation (CV) values of 6.55, 8.17, 6.56 and 7.93% for LTN, RTN, TNUM and MAXAP, respectively.

In Table 1, it was shown that the values of SNP-based heritability ($h_{\mathrm{SNP}}^{2}$) for teat number-related traits were 0.29, 0.19, 0.36, 0.38, 0.00 and 0.24 for LTN, RTN, TNUM, MAXAP, L-R and ADIFF in Qingping pigs, respectively. Unfortunately, the standard errors (SE) were large, which reflects the low accuracy of the heritability estimates. Therefore, we compared the results of the teat number-related heritability estimates in Qingping pigs with those of other pig breeds. In Qingping pigs, the narrow-sense heritability for TNUM (0.36) (only considering the contribution of additive genetic effects) was consistent with the values of purebred Korean Yorkshire pigs (0.37) [4] and Duroc pigs (0.34 ± 0.05) [3]. In previous studies, the $h_{\mathrm{SNP}}^{2}$ values of LTN, RTN, MAXAP and L-R were 0.16, 0.26, 0.261 and 0.00, which were consistent with our results [17]. Unexpectedly, ADIFF had a medium $h_{\mathrm{SNP}}^{2}$ value (0.24), which was virtually null in previous studies [16,17]. These results suggest that estimates of the heritability of teat number-related traits in Qingping pigs were reliable, but caution is required due to the large standard errors.

### 3.2. GLM GWAS for Teat Number-Related Traits

PCA results indicated that Qingping pigs could not be clustered into groups (Figure S1), so GWAS was performed using GLM with no principal component. The Bonferroni correction assumes that each of the tests is independent, an is thereby inherently conservative when considering SNPs in LD. We calculated the effectively independent tests based on the estimated number of independent markers [32]. A total of 557,540 SNP and 28,629 Indel independent tests were suggested, with the threshold *p*-value of 8.97 × 10^−8^ (0.05/557,540) for SNPs and 1.75 × 10^−6^ (0.05/28,629) for Indels.

**Figure 2:**
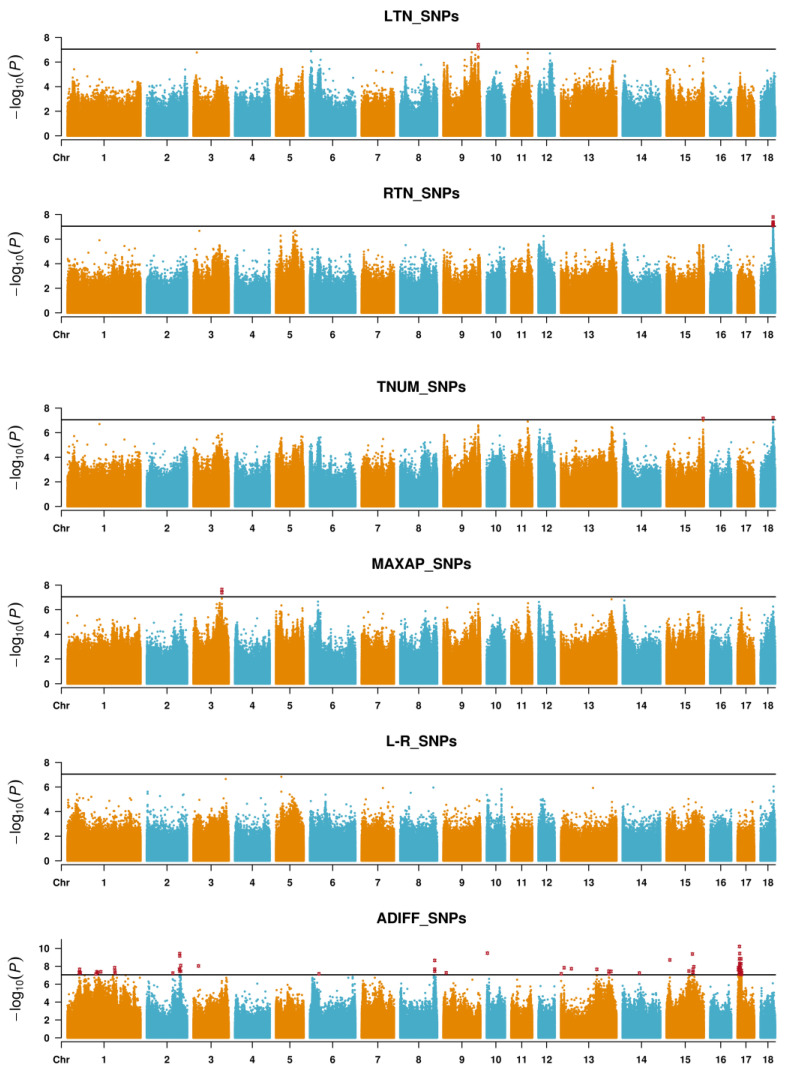
Manhattan plots of GLM GWAS for teat number-related traits in Qingping pigs, including LTN, RTN, TNUM, MAXAP, L-R and ADIFF based on SNPs. LTN: the number of teats on the left side; RTN: the number of teats on the right side; TNUM: the total number of teats (TNUM = LTN + RTN); MAXAP: the maximum number of teats in LTN and RTN (MAXAP); L-R: the difference between the two sides (L-R = LTN − RTN); ADIFF: the absolute difference between left and right teat number (ADIFF = |LTN − RTN|).

**Figure 3:**
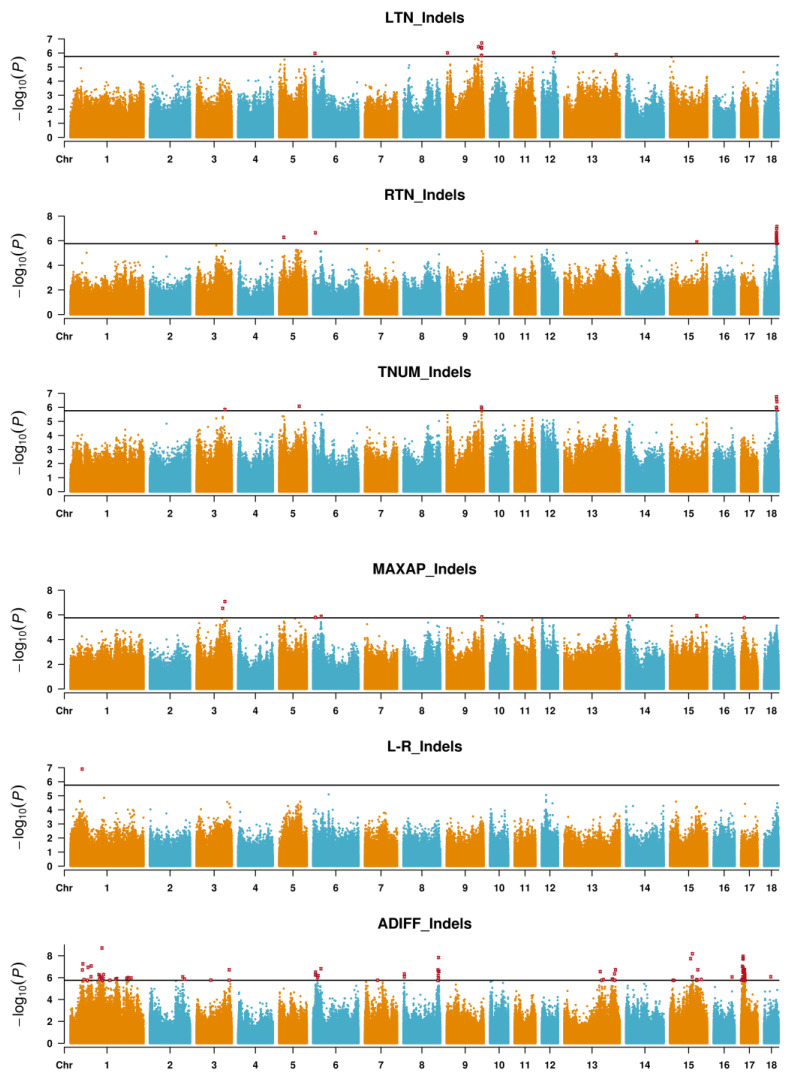
Manhattan plots of GLM GWAS for teat number-related traits in Qingping pigs, including LTN, RTN, TNUM, MAXAP, L-R and ADIFF based on Indels. LTN: the number of teats on the left side; RTN: the number of teats on the right side; TNUM: the total number of teats (TNUM = LTN + RTN); MAXAP: the maximum number of teats in LTN and RTN (MAXAP); L-R: the difference between the two sides (L-R = LTN − RTN); ADIFF: the absolute difference between left and right teat number (ADIFF = |LTN − RTN|).

Figure 2 and Figure 3 show the Manhattan plots for LTN, RTN, TNUM, MAXAP, L-R and ADIFF, and Figure S2 presents the Q-Q plots for these traits. A total of 28 significant SNPs and 45 significant Indels were detected as peak associated variants for LTN, RTN, TNUM, MAXAP, L-R and ADIFF (Table 2). Among the 28 SNPs identified, 10 SNPs were located within 11 genes and the others (18 SNPs) were located at 3798 to 46,070,668 bp from the nearest genes (Table 2). Among the 45 Indels identified, 21 Indels were located within 22 genes and the others (24 Indels) were located 2660 to 137,303 bp from the nearest genes (Table 2).

Compared with known QTLs for teat number-related traits in the PigQTL database, 14 significant SNPs and 24 significant Indels overlapped with known QTLs (Table 2). Interestingly, Manhattan plots showed similar trends between SNPs and Indels. In Table 2, there were several significant SNPs and Indels were located at the same teat number-related traits QTLs in the PigQTL database. For RTN, significant SNP (rs345573243) and Indel (chr18:48316684) on SSC18 were located at QTL 7470 and QTL 24290, and coincidentally, rs345573243 and chr18:48316684 also showed significant associations with TNUM. For MAXAP, significant SNP (rs703282466) and Indel (rs793312568) on SSC3 were located at QTL 4250 and 4256, and coincidentally, rs793312568 also exhibited a significant association with TNUM. For ADIFF, significant SNPs (rs326371568 and rs342451777) and Indel (rs1113667849) on SSC2 were located at QTL 4255; significant SNP (rs701874475) and Indel (chr13:134712100) on SSC13 at QTL 7479; significant SNP (rs326978910) and Indel (chr15:84277014) on SSC15 at QTL 7468. Additionally, five new significant SNPs were closed to significant Indels, including rs1108940033 was closed to chr1:44973455 and chr1:46840905 on SSC1, rs338649298 was closed to chr8:131927486 on SSC8, rs343864506 was closed to chr13:188309252 on SSC13, rs1109225784 was closed to chr15:12585471 on SSC15, chr17:8221026 was closed to rs700363122 on SSC17. 

### 3.3. FarmCPU GWAS for ADIFF

**Figure 4:**
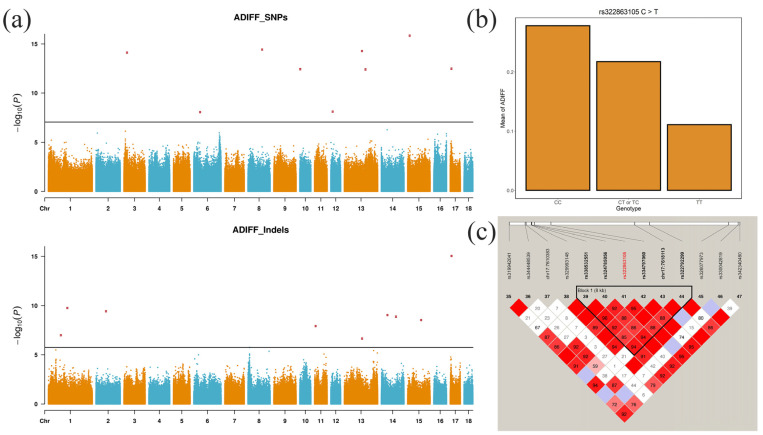
FarmCPU GWAS for ADIFF. (**a**) Manhattan plots of the GWAS based on SNPs and Indels. (**b**) Difference analysis of the strongest significant SNP (rs322863105) on SSC17 in GLM, which was retested in FarmCPU. (**c**) Haplotype block analysis of selected suggestive significant SNPs associated with ADIFF on SSC17 in GLM, including rs322863105. ADIFF: the absolute difference between left and right teat number (ADIFF = |LTN − RTN|).

The GLM GWAS results of ADIFF showed significant associations even using whole genome SNPs or Indels to decide the thresholds (SNP: 0.05/2,319,3931; Indels: 0.05/2,053,221). However, Q-Q plots and genomic inflation factors (λ_SNP_ = 1.37, λ_Indel_ = 1.36) indicated possible false positives (Figure S2). FarmCPU, a powerful and efficient GWAS model, was used to control false positives and retain true positives. Q-Q plots and genomic inflation factors (λ_SNP_ = 0.98, λ_Indel_ = 0.86) were improved by FarmCPU (Figure S3). In Figure 4a and Table 3, 9 SNPs and 9 Indels were shown to be significantly associated variants for ADIFF on SSC1, 2, 3, 6, 8, 10, 11, 12, 13, 14, 15 and 17, with six SNPs and five Indels included in known QTLs (Table 3).

Three of the 9 significant SNPs were located within four genes and six SNPs were located 15,848 to 133,156 bp from the nearest genes (Table 3). Three of the 9 significant Indels were located within three genes and six Indels were located 3267 to 99,873 bp from the nearest genes (Table 3). Compared with the GLM GWAS results for ADIFF, 4 SNPs (rs325963999, rs1109963100, rs1109225784, rs322863105) and four Indels (chr1:77875820, chr1:118273285, chr15:84277014, rs700363122) were duplicated in the FarmCPU GWAS results, suggesting the reliability of the results (Table 3).

The strongest significant SNP in GLM for ADIFF on SSC17 (rs322863105, *p*-value = 6.01 × 10^−^^11^) also showed significant association with ADIFF in FarmCPU (*p*-value = 3.34 × 10^−^^13^). The effect of rs322863105 on the ADIFF was estimated by genotyping Qingping pigs for this SNP. Individuals with the TT genotype had a lower ADIFF, suggesting LTN and RTN were more symmetrical (Figure 4b). Linkage analysis of the suggestive significant SNPs around this SNP identified one haplotype block of 8 kb between rs338532551 and rs322792299, including rs322863105 (Figure 4c). Three annotated genes were contained in the 1-Mb region around rs322863105, including *tripartite motif family like 1* (*TRIML1*), *tripartite motif family like 2* (*TRIML2*), and *ZFP42 zinc finger protein* (*ZFP42*). Moreover, a peak Indel (rs700363122) close to this SNP showed significant associations with ADIFF in the results of both GLM (1.13 × 10^−^^8^) and FarmCPU (9.10 × 10^−^^16^), with two annotated genes in the 1-Mb region around this Indel, including *FAT Atypical Cadherin 1* (*FAT1*), and *Melatonin Receptor 1A* (*MTNR1A*).

### 3.4. Functional Enrichment of Candidate Genes

**Figure 5:**
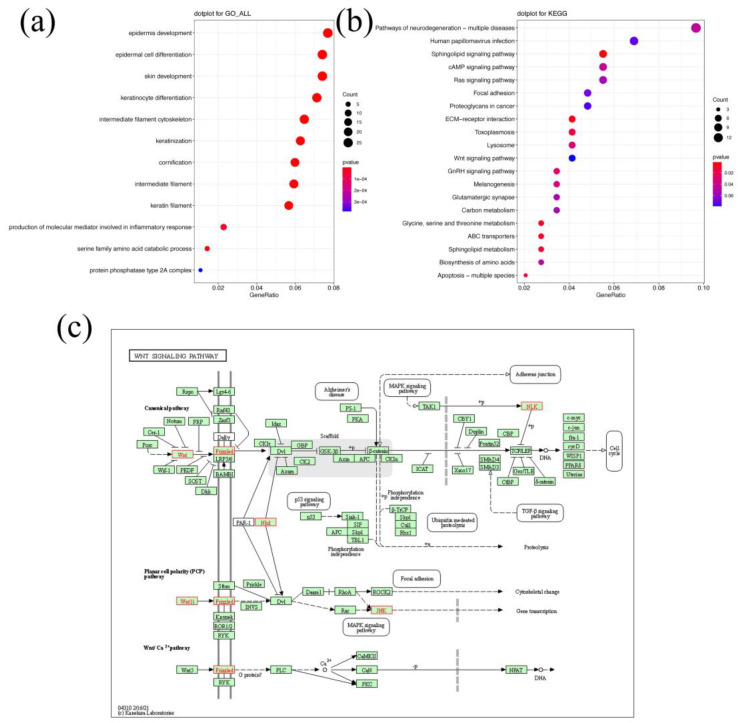
Enrichment results of candidate genes within 1-Mb regions of significant SNPs and Indels. (**a**) Dotplot of GO term enrichment. (**b**) Dotplot of KEGG pathway enrichment. (**c**) Wnt signaling pathway.

Annotated genes within 1-Mb regions of significant SNPs and Indels were defined as candidate genes. A total of 397 annotated genes were found in these regions (Table S2). In Figure 5a, GO enrichment analysis showed the enrichment of these candidate genes in epidermis development (*p* = 2.31 × 10^−^^7^), epidermal cell differentiation (*p* = 4.61 × 10^−^^9^), and skin development (*p* = 1.12 × 10^−^^7^). In Figure 5b KEGG pathway analysis revealed the enrichment of candidate genes in the pathways, such as the Sphingolipid signaling pathway (*p* = 1.27 × 10^−^^3^), ECM-receptor interaction (*p* = 4.79 × 10^−^^3^), and Glycine, serine and threonine metabolism (*p* = 5.43 × 10^−^^3^). Furthermore, we also paid attention to the Wnt signaling pathway (Figure 5c), due to its important role in initiating mammary morphogenesis and all subsequent stages of mammary formation as previously reported [33].

## 4. Discussion

During the first month after birth, piglets only have sow milk as a source of nutrients, which contributes to the regulation of their basal metabolism and temperature. Therefore, the sow’s ability to produce milk can influence the health and growth of piglets, probably with a long-term effect post-weaning. The sows’ lactation performance can be improved by enhancing the growth of the mammary gland and sows with a low prolactin/progesterone ratio before farrowing were reported to have a higher colostrum yield [34]. Additionally, milk production could be increased by adding L-arginine to the diets of lactating primiparous sows [35]. Moreover, for primiparous sows, teat suckling only for the first 2 days postpartum ensures the optimal mammary development and milk yield in the next lactation [36]. Furthermore, the lactating ability of sows can also be improved by increasing the teat number. As shown in the present study, teat number is heritable, with a genetic correlation to numerous microsatellite sites and SNPs (especially a QTL on SSC7).

The $h_{\mathrm{SNP}}^{2}$ of most teat number-related traits in Qingping pigs was moderate, except for L-R, suggesting it is feasible to improve teat number in pigs through genetic selection. SNPs and Indels associated with teat number-related traits might play an essential role in teat number improvement. Several candidate genes were reported to be related to mammary gland development and breast cancer. *CDYL2*, including an Indel significantly associated with LTN on SSC6, positively regulates breast cancer cell migration, invasion and epithelial-to-mesenchymal transition through p65/NF-κB and STAT3 [37]. *FAM3C*, which is in the 1-Mb region of an Indel on SSC18 and associated with ADIFF, encodes Interleukin-Like Epithelial-Mesenchymal Transition Inducer for the proliferation and migration of breast cancer cells [38]. *WWOX*, including an Indel (chr6:9186279) significantly associated with RTN, is known to play a role in breast cancer [39]. *TRIML2* and *MTNR1A* were close to SNP (rs330045817) and Indel (rs700363122), respectively, on SSC17. As mentioned above, these two different types of variants were close to each other and significantly associated with ADIFF in both GLM and FarmCPU models. *TRIML2* was significantly associated with prognosis, with a higher expression in triple-negative breast cancer cell lines than in normal mammary cell lines [40]. Common variants in *MTNR1A* may contribute to breast cancer susceptibility [41]. *TBX5*, encoding *T-Box Transcription Factor 5*, was close to the significant Indel (rs701717756) for ADIFF on SSC14. In a large German family, *TBX3* and *TBX5* duplication was reported to be associated with Ulnar-Mammary syndrome [42]. Importantly, *TBX3* was the placode marker required for the formation of mammary placodes and the development of fetal mammary glands in all mammals [43]. Although not located in the 1-Mb region of rs701717756, *TBX3* was close to this Indel with a distance of 625,516 bp.

Unlike earlier studies, the present study failed to detect significant association signals in genome regions around *VRTN* on SSC7, which was reported as a credible candidate gene for the teat number and the vertebra number [17,44]. Zhuang et al. suggested that the genetic heterogeneity of variants in *VRTN* may exist in different populations and *VRTN* may not be a strong or the only causal gene for teat number based on their finding that *VRTN* mutation was significantly associated with the teat number in Canadian Duroc pigs, but not in American Duroc pigs [14]. Moreover, *VRTN* mutation on SSC7 was also not significantly associated with the teat number in Chinese pig breeds, including Beijing Black pig and Sushan pig [8,9], but significantly associated with the vertebra number in Beijing Black pig. Furthermore, *VRTN* was reported to modulate somite segmentation [44]. These reports suggested that *VRTN* plays a more important role in vertebra number and varies in its role in teat number among populations with different genetic backgrounds. Therefore, the teat number in Qingping pigs is speculated to involve other genes or pathways.

The GO enrichment results showed significant enrichment in skin development, epidermis development, and epidermal cell differentiation. The mammary gland is an epithelial organ, and epithelial–stromal crosstalk is a key aspect of mammary morphogenesis [45]. In KEGG enrichment analysis, the Wnt signaling pathway did not reach a significance level of 0.05 (0.078). Interestingly, candidate genes, including *WNT11*, *WNT16* and *FZD3*, were located at key positions in this pathway (Figure 5c). *WNT16* and *FZD3* were also involved in GO terms, skin development and epidermis development. The Wnt signaling cascade is implicated in almost all stages of mammary development and is pivotal for the specification and morphogenesis of the mammary gland [46]. *WNT11* was expressed in stromal cells and basal cells in the adult mammary gland [46]. Chu et al. reported that *WNT11* and *FZD3* were expressed in mammary buds at E12.5 and E15.5 [47]. Importantly, Wnt signaling interacted with *TBX3* in mammary placode development [47]. These reports suggested that candidate genes might affect teat number during mammary gland morphogenesis. Unlike other breeds, the teat number of Qingping pigs showed a medium heritability (0.24) for ADIFF, implying the potential involvement of candidate genes in mammary gland morphogenesis. Therefore, our candidate genes related to mammary gland morphogenesis and development can be assumed to contribute to teat number improvement and even may influence milk production.

## 5. Conclusions

In this study, GLM and FarmCUP GWAS were carried out to detect associated SNPs and Indels for 6 teat number-related traits. We found a total of 33 SNPs and 50 Indels for teat number. The most significant SNP and Indel were located on SSC17. Six candidate genes were enriched in the Wnt signaling pathway with an important role in mammary gland morphogenesis and development. A novel candidate gene on SSC14, *TBX3*, was detected as a mammary placode marker. These findings contribute to our understanding of the genetic architecture of teat number and provide genetic markers for genetic improvement of teat number in Qingping pigs.

## Figures and Tables

**Table 1:** Summary of phenotypic data in terms of mean, standard deviation, minimum, maximum, coefficient of variance, and SNP-based heritability and standard error (SE) for each trait.

Trait	N	Mean	SD	Min	Max	C.V.	$h_{\mathrm{SNP}}^{2}$	SE
LTN	100	7.36	0.48	7.00	8.00	6.55	0.29	0.21
RTN	100	7.42	0.61	6.00	9.00	8.17	0.19	0.21
TNUM	100	14.78	0.97	14.00	17.00	6.56	0.36	0.23
MAXAP	100	7.50	0.59	7.00	9.00	7.93	0.38	0.22
L-R	100	−0.06	0.51	−2.00	2.00	NA	0.00	0.20
ADIFF	100	0.22	0.46	0.00	2.00	NA	0.24	0.20

LTN: the number of teats on the left side; RTN: the number of teats on the right side; TNUM: the total number of teats (TNUM = LTN + RTN); MAXAP: the maximum number of teats in LTN and RTN (MAXAP); L-R: the difference between the two sides (L-R = LTN − RTN); ADIFF: the absolute difference between left and right teat number (ADIFF = |LTN − RTN|).

**Table 2:** Significant SNPs and Indels of GLM GWAS for teat number-related traits.

SNP	Trait	SSC	Position	Effect	*p*-Value	QTLs *	Annotation	Gene (Distance from the Gene in bp)
chr9:130956708	LTN	9	130956708	0.64	3.85 × 10^−^^8^		intronic	*PACC1*(*within*)*, NENF*(*within*)
rs345573243	RTN	18	47399908	0.62	1.60 × 10^−^^8^	24,290, 7470	intronic	*OSBPL3(within)*
rs703282466	MAXAP	3	106788730	0.45	2.29 × 10^−^^8^	5224, 8797, 8798, 4250, 4256	intergenic	*LTBP1(63796), ENSSSCG00000050704(9889)*
chr15:137183045	TNUM	15	137183045	−0.79	7.02 × 10^−^^8^	223,293	intronic	*MLPH(within)*
rs345573243	TNUM	18	47399908	0.96	6.25 × 10^−^^8^	24,290, 7470	intronic	*OSBPL3(within)*
rs1108940033	ADIFF	1	45796983	0.61	2.19 × 10^−^^8^		intergenic	*PHF3(500583),ENSSSCG00000049526(273685)*
rs321204530	ADIFF	1	177056841	0.59	1.44 × 10^−^^8^	5223, 5255, 822, 845, 1250	intronic	*MDGA2(within)*
rs318957512	ADIFF	2	97849348	0.50	5.79 × 10^−^^8^		intronic	*ADGRV1(within)*
rs326371568	ADIFF	2	123586226	0.62	3.87 × 10^−^^10^	4255	intergenic	*FAM170A(119517),PRR16(613282)*
rs342451777	ADIFF	2	127224122	0.57	8.31 × 10^−^^9^	4255	intergenic	*ENSSSCG00000042143(13463),ENSSSCG00000040936(55836)*
rs325963999	ADIFF	3	19301837	0.66	9.15 × 10^−^^9^	5224, 7455, 7472	intronic	*KATNIP(within)*
rs326276043	ADIFF	6	34051848	0.55	6.98 × 10^−^^8^	24,289	intergenic	*ENSSSCG00000050973(273191),CYLD(8093)*
rs338649298	ADIFF	8	129552162	0.59	2.22 × 10^−^^9^		intergenic	*SNCA(163855),ENSSSCG00000043431(95940)*
rs321470648	ADIFF	9	10769337	0.45	5.38 × 10^−^^8^		intergenic	*ENSSSCG00000046278(36887),ENSSSCG00000045225(26362)*
rs1109963100	ADIFF	10	2911179	0.80	3.37 × 10^−^^10^		intergenic	*ENSSSCG00000042899(210778),BRINP3(52348)*
rs339887165	ADIFF	13	12135388	0.68	1.46 × 10^−^^8^	7479	intergenic	*ENSSSCG00000044771(16544),ENSSSCG00000051554(31818)*
chr13:39266305	ADIFF	13	39266305	0.72	1.88 × 10^−^^8^	7479	intronic	*DNAH12(within)*
rs701874475	ADIFF	13	134665423	−0.51	2.19 × 10^−^^8^	7479	intergenic	*LMLN(8675),ENSSSCG00000050583(21280)*
rs338558804	ADIFF	13	179683904	−0.45	3.49 × 10^−^^8^		intergenic	*ENSSSCG00000038062(120975),NRIP1(140958)*
rs343864506	ADIFF	13	187708682	−0.54	3.72 × 10^−^^8^		intergenic	*ENSSSCG00000047308(444803),ENSSSCG00000050420(500018)*
rs334271954	ADIFF	14	62959726	0.52	5.70 × 10^−^^8^		intergenic	*FAM13C(33877),SLC16A9(66937)*
rs1109225784	ADIFF	15	12581324	0.64	1.96 × 10^−^^9^		intergenic	*U6(133156),U6(186459)*
rs326978910	ADIFF	15	84015934	0.38	3.38 × 10^−^^8^	7468	intronic	*OSBPL6(within)*
rs334746473	ADIFF	15	97200323	0.75	4.23 × 10^−^^10^	7468	intergenic	*ENSSSCG00000046205(186079),U2(688281)*
rs322863105	ADIFF	17	7610979	−0.88	6.01 × 10^−^^11^		intergenic	*ENSSSCG00000045345(132678),ENSSSCG00000047202(438984)*
chr17:8221026	ADIFF	17	8221026	0.79	1.47 × 10^−^^9^		intergenic	*U6(19092),FAT1(227734)*
rs330045817	ADIFF	17	8536301	−0.71	3.76 × 10^−^^10^		ncRNA_intronic	*FAT1(within)*
rs324534432	ADIFF	17	13364668	0.79	1.47 × 10^−^^9^		intergenic	*PSD3(100469),ENSSSCG00000046441(3798)*
Indel								
chr6:7472906	LTN	6	7472906	−0.41	1.06 × 10^−^^6^	24,289	intronic	*CDYL2(within)*
rs695882779	LTN	9	3690507	0.39	9.84 × 10^−^^7^		ncRNA_intronic	*ENSSSCG00000049604(within)*
chr9:119650540	LTN	9	119650540	−0.30	3.62 × 10^−^^7^		intergenic	*ENSSSCG00000044083(337170),ENSSSCG00000050832(4698)*
rs792699200	LTN	9	130899869	0.51	4.41 × 10^−^^7^		intronic	*PACC1(within)*
chr12:44292044	LTN	12	44292044	−0.37	9.55 × 10^−^^7^	5227, 5261, 6472, 6479, 595, 2929	intronic	*NOS2(within)*
chr13:194617904	LTN	13	194617904	−0.31	1.30 × 10^−^^6^		intergenic	*KRTAP11-1(111382),ENSSSCG00000047315(5813)*
rs709659410	RTN	5	17969771	0.42	5.28 × 10^−^^7^	2927	intergenic	*KRT73(2682),KRT2(14544)*
chr6:9186279	RTN	6	9186279	0.89	2.28 × 10^−^^7^	24,289	intronic	*WWOX(within)*
rs790747253	RTN	15	100810568	−0.61	1.28 × 10^−^^6^	7468	intronic	*PGAP1(within)*
chr18:48316684	RTN	18	48316684	0.41	7.00 × 10^−^^8^	24,290, 7470	intronic	*STK31(within)*
chr3:98429885	MAXAP	3	98429885	0.50	2.93 × 10^−^^7^	5224, 8797, 8798	intergenic	*ENSSSCG00000045166(59604),ENSSSCG00000046007(449247)*
rs793312568	MAXAP	3	106792080	0.43	8.28 × 10^−^^8^	5224, 8797, 8798, 4250, 4256	intergenic	*LTBP1(67153),ENSSSCG00000050704(6530)*
chr6:9186279	MAXAP	6	9186279	0.82	1.59 × 10^−^^6^	24,289	intronic	*WWOX(within)*
chr6:30642639	MAXAP	6	30642639	0.43	1.31 × 10^−^^6^	24,289	intergenic	*ENSSSCG00000047270(70049),ENSSSCG00000041426(189382)*
chr9:131996847	MAXAP	9	131996847	−0.49	1.46 × 10^−^^6^		intronic	*ENSSSCG00000040650(within)*
chr14:12750600	MAXAP	14	12750600	0.43	1.31 × 10^−^^6^		intronic	*HMBOX1(within)*
rs790747253	MAXAP	15	100810568	−0.60	1.17 × 10^−^^6^	7468	intronic	*PGAP1(within)*
rs711984029	MAXAP	17	13041965	−0.47	1.68 × 10^−^^6^		intronic	*PSD3(within)*
rs793312568	TNUM	3	106792080	0.63	1.41 × 10^−^^6^	5224, 8797, 8798, 4250, 4256	intergenic	*LTBP1(67153),ENSSSCG00000050704(6530)*
chr5:75592729	TNUM	5	75592729	0.98	8.38 × 10^−^^7^		intronic	*NELL2(within)*
rs792699200	TNUM	9	130899869	1.00	9.67 × 10^−^^7^		intronic	*PACC1(within)*
chr18:48316684	TNUM	18	48316684	0.62	4.01 × 10^−^^7^	24,290, 7470	intronic	*STK31(within)*
chr1:44096236	LR	1	44096236	0.69	1.25 × 10^−^^7^		intergenic	*ENSSSCG00000042072(159101),ENSSSCG00000045405(51729)*
chr1:44973455	ADIFF	1	44973455	0.70	1.96 × 10^−^^7^		intronic	*ZUP1(within),RSPH4A(within)*
chr1:46840905	ADIFF	1	46840905	0.71	5.46 × 10^−^^8^		intergenic	*ENSSSCG00000050391(123491),U6(392319)*
chr1:65957275	ADIFF	1	65957275	0.43	1.14 × 10^−^^7^		intergenic	*FBXL4(21578),FAXC(274880)*
chr1:77875820	ADIFF	1	77875820	0.42	8.57 × 10^−^^8^		intergenic	*FYN(65243),U6(107381)*
chr1:118273285	ADIFF	1	118273285	−0.64	1.96 × 10^−^^9^	5223, 6481, 5255	intergenic	*ENSSSCG00000049391(9019),ENSSSCG00000045826(5556)*
rs1113667849	ADIFF	2	123584099	0.55	8.50 × 10^−^^7^	4255	intergenic	*FAM170A(117391),PRR16(615405)*
chr3:54080763	ADIFF	3	54080763	0.44	1.70 × 10^−^^6^	5224, 7455, 7472, 6465	intergenic	*LONRF2(99093),REV1(54761)*
chr3:122749868	ADIFF	3	122749868	0.76	1.91 × 10^−^^7^		intergenic	*LRATD1(17941),ENSSSCG00000045589(179131)*
chr6:9702570	ADIFF	6	9702570	0.61	3.15 × 10^−^^7^	24,289	intronic	*WWOX(within)*
chr6:29725029	ADIFF	6	29725029	−0.41	1.54 × 10^−^^7^	24,289	intergenic	*ENSSSCG00000034192(132519),CES5A(117783)*
chr7:48226837	ADIFF	7	48226837	0.67	1.74 × 10^−^^6^	5257	intronic	*RASGRF1(within)*
chr8:3878010	ADIFF	8	3878010	−0.48	4.61 × 10^−^^7^	7477	intronic	*ENSSSCG00000027349(within)*
chr8:131927486	ADIFF	8	131927486	0.68	1.46 × 10^−^^8^		intergenic	*AFF1(2666),ENSSSCG00000032190(41799)*
chr13:134712100	ADIFF	13	134712100	−0.50	2.83 × 10^−^^7^	7479	intergenic	*ENSSSCG00000050583(20384),OSBPL11(6327)*
chr13:188309252	ADIFF	13	188309252	0.35	4.51 × 10^−^^7^		intergenic	*ENSSSCG00000050420(95234),ENSSSCG00000043493(500979)*
chr13:191714830	ADIFF	13	191714830	0.76	1.91 × 10^−^^7^		intergenic	*ENSSSCG00000051384(248028),ENSSSCG00000048685(53357)*
chr15:12585471	ADIFF	15	12585471	0.67	1.74 × 10^−^^6^		intergenic	*U6(137304),U6(182308)*
chr15:84277014	ADIFF	15	84277014	0.53	6.57 × 10^−^^9^	7468	intergenic	*ENSSSCG00000036052(46519),ENSSSCG00000038561(122000)*
rs792656057	ADIFF	16	69550278	−0.60	8.69 × 10^−^^7^	5228	intronic	*GRIA1(within)*
rs793561441	ADIFF	17	5622328	−0.33	9.14 × 10^−^^8^		intronic	*PCM1(within)*
rs700363122	ADIFF	17	8173767	0.60	1.13 × 10^−^^8^		intergenic	*ENSSSCG00000047202(116838),U6(28063)*
rs789477433	ADIFF	18	25541841	0.43	8.35 × 10^−^^7^	24,290	intergenic	*ENSSSCG00000048651(270433),FAM3C(27695)*

* QTL number in PigQTL database. Effect means additive effect. LTN: the number of teats on the left side; RTN: the number of teats on the right side; TNUM: the total number of teats (TNUM = LTN + RTN); MAXAP: the maximum number of teats in LTN and RTN (MAXAP); L-R: the difference between the two sides (L-R = LTN − RTN); ADIFF: the absolute difference between left and right teat number (ADIFF = |LTN − RTN|).

**Table 3:** Significant SNPs and Indels of FarmCPU GWAS for ADIFF.

SNP	SSC	Position	Effect	*p*-Value	QTLs *	Annotation	Gene (Distance from the Gene in bp)
rs325963999 ^#^	3	19301837	0.27	7.82 × 10^−^^15^	5224, 7455, 7472	intronic	*KATNIP(within)*
rs693622708	6	39540583	0.17	8.58 × 10^−^^9^	24,289	intergenic	*UQCRFS1(166710), ENSSSCG00000050718(41760)*
rs326134805	8	88543901	0.13	3.81 × 10^−^^15^	7477, 1100	intergenic	*ENSSSCG00000044017(86061),SLC7A11(63983)*
rs1109963100 ^#^	10	2911179	0.33	3.74 × 10^−^^13^		intergenic	*ENSSSCG00000042899(210778),BRINP3(52348)*
rs1113875395	12	11463144	0.16	7.51 × 10^−^^9^	5227, 1128	intergenic	*ABCA8(15848),ENSSSCG00000045738(20026)*
rs343773900	13	110154062	0.24	5.42 × 10^−^^15^	7479	intronic	*PLD1(within)*
rs333970515	13	132158230	−0.18	3.99 × 10^−^^13^	7479	ncRNA_exonic	*ENSSSCG00000047632(within)*
rs1109225784 ^#^	15	12581324	0.32	1.46 × 10^−^^16^		intergenic	*U6(133156),U6(186459)*
rs322863105 ^#^	17	7610979	−0.34	3.34 × 10^−^^13^		intergenic	*ENSSSCG00000045345(132678),ENSSSCG00000047202(438984)*
Indel							
chr1:77875820 ^#^	1	77875820	0.17	1.02 × 10^−^^7^		intergenic	*FYN(65243),U6(107381)*
chr1:118273285 ^#^	1	118273285	−0.35	1.74 × 10^−^^10^	5223, 6481, 5255	intergenic	*ENSSSCG00000049391(9019),ENSSSCG00000045826(5556)*
rs788352632	2	62598411	−0.12	3.81 × 10^−^^10^	909	ncRNA_intronic	*ENSSSCG00000048292(within)*
chr11:6896071	11	6896071	−0.13	1.22 × 10^−^^8^	5260	ncRNA_intronic	*ENSSSCG00000036846(within)*
rs787621311	13	110898097	0.23	2.27 × 10^−^^7^	7479	intronic	*FNDC3B(within)*
rs701717756	14	38222283	0.26	9.42 × 10^−^^10^		intergenic	*RBM19(17536),ENSSSCG00000042669(3247)*
chr14:91416556	14	91416556	−0.30	1.34 × 10^−^^9^		intergenic	*ENSSSCG00000047278(204580),CXCL12(99865)*
chr15:84277014 ^#^	15	84277014	0.22	2.92 × 10^−^^9^	7468	intergenic	*ENSSSCG00000036052(46519),ENSSSCG00000038561(122000)*
rs700363122 ^#^	17	8173767	0.40	9.10 × 10^−^^16^		intergenic	*ENSSSCG00000047202(116838),U6(28063)*

* QTL number in in PigQTL database. ^#^ Duplicate signals between GLM and FarmCPU. Effect means additive effect. ADIFF: the absolute difference between left and right teat number (ADIFF = |LTN − RTN|).

## Data Availability

All raw sequences for the 100 QP pigs have been deposited into the NCBI Sequence Read Archive under PRJNA489520 and will be available on 30 December 2023. Phenotypes and genotypes are deposited in OSF of Center for Open Science (https://osf.io/szu86/) (will be accessible on 30 December 2023).

## Supplementary Materials

The following supporting information can be downloaded at: https://www.mdpi.com/article/10.3390/ani12091057/s1. Figure S1: Principal component analysis of Qingping pigs; Figure S2: The Q-Q plots of the GLM GWAS for teat number-related traits in Qingping pigs, including LTN, RTN, TNUM, MAXAP, L-R and ADIFF. (a–f) The Q-Q plots of GLM GWAS based on SNPs. (g–l) The Q-Q plots of GLM GWAS based on Indels; Figure S3: The Q-Q plots of the FarmCPU GWAS for ADIFF in Qingping pigs. (a) The Q-Q plot of FarmCPU GWAS based on SNPs. (b) The Q-Q plot of FarmCPU GWAS based on Indels; Table S1: Sequencing reads, alignment statistics, and mean coverage for each sample in this study; Table S2: Annotated genes within 1-Mb regions of significant SNPs and Indels.
