# Supplementary material for: A Whole Genome Sequencing-Based Genome-Wide Association Study Reveals the Potential Associations of Teat Number in Qingping Pigs
Source: Animals (Basel). 2022 Apr 20;12(9):1057. doi: 10.3390/ani12091057 (PMC9100799; doi:10.3390/ani12091057)
Supplement: Supplementary file 1 [file animals-12-01057-s001.zip › animals-1605502-supplementary.pdf]

A whole genome sequencing-based genome-wide association study reveals the potential associations of teat number in Qingping pigs

Zezhang Liu 1, Hong Li 2, Zhuxia Zhong 1 and Siwen Jiang 1,*

Supporting Information

Table S1 Sequencing reads, alignment statistics, and mean coverage for each sample in this study.

Sample	Raw data(bp)	Clean data(bp)	Rate of GC(%)	Mapping rate(%)	Average depth
**1028**	27,601,120,500	27,312,580,500	44.56	98.01	9.38
**1038**	26,329,410,900	26,187,763,800	43.48	98.51	9.41
**1050**	31,247,861,700	30,959,790,000	44.17	98.76	10.86
**1052**	30,423,788,400	30,230,559,600	44.62	98.49	10.37
**1068**	30,746,581,200	30,635,812,500	42.92	98.86	10.97
**1084**	30,905,747,700	30,648,904,200	44.72	98.59	10.91
**1086**	28,607,019,600	28,361,117,700	44.8	98.41	10.15
**1088**	26,754,777,300	26,619,815,700	44.31	98.42	9.05
**1102**	26,331,194,400	26,184,072,300	43.82	98.2	8.92
**1104**	28,180,245,600	27,997,597,800	43.39	98.11	10.08
**1108**	29,526,411,900	29,304,994,200	44.3	98.32	10.02
**1110**	27,820,550,700	27,657,441,900	44.78	98.7	9.7
**1132**	26,846,375,100	26,551,257,600	45.06	98.43	9.53
**1138**	27,562,471,800	27,399,692,100	43.28	98.47	9.68
**1140**	26,391,909,600	26,240,262,000	43.75	98.88	9.44
**1152**	28,458,898,800	28,394,755,200	44.69	98.76	10.04
**1160**	29,234,153,100	29,128,874,400	43.8	98.84	10.43
**1180**	25,401,510,600	25,051,094,700	45.8	98.11	8.94
**1186**	25,585,786,800	25,374,612,900	44.12	98.35	9.04
**1190**	26,356,383,900	26,115,585,900	46.72	97.95	9.39
**1204**	29,175,401,700	28,812,598,500	43.84	98.55	10.07
**1206**	29,372,100,300	29,073,833,400	44.02	98.69	10.13
**1208**	28,305,269,400	28,141,845,600	42.74	98.87	9.68
**1602**	26,665,902,900	26,514,654,300	42.39	98.49	9.25
**1710**	26,508,871,800	26,414,685,900	42.36	98.57	9.15
**1718**	25,806,428,100	25,546,341,000	44.81	98.06	9.16
**1810**	27,960,520,200	27,653,754,600	44.67	98.56	9.94
**2006**	29,304,964,500	28,977,112,500	45.33	98.31	9.96
**2008**	26,114,335,200	25,770,977,700	46.34	98.44	9.11
**2028**	26,301,945,900	26,152,471,800	45.48	98.58	9.36
**2030**	24,192,264,600	24,008,830,500	44.81	98.68	8.53
**2034**	29,269,947,300	29,078,745,000	44.92	98.55	9.95
**2036**	26,085,227,700	25,534,313,100	44.5	98.74	8.8
**2050**	27,817,381,800	27,387,529,200	43.84	98.81	9.62
**2052**	28,331,300,400	28,119,179,100	43.64	98.63	10.03
**2090**	28,149,704,700	27,870,952,200	45.54	98.3	9.69
**2300**	30,879,943,800	30,562,385,400	43.56	98.73	10.8
**2314**	27,952,674,300	27,810,540,900	43.08	99.03	9.83
**2344**	27,941,992,200	27,757,090,200	43.8	98.42	9.91
**2346**	26,142,688,200	25,801,932,300	44.95	98.39	8.97
**2400**	26,667,749,400	26,478,414,000	44.22	98.27	9.47
**2402**	26,648,643,300	26,388,661,500	45.34	98.45	9.3
**3000**	27,773,948,400	27,321,546,600	45.78	98.29	9.82
**3008**	25,695,036,300	25,510,958,400	43.78	98.37	9.17
**3032**	25,171,536,600	24,624,036,900	43.62	98.81	8.64
**3034**	29,751,275,100	29,554,689,300	44.44	98.49	10.21
**3052**	31,825,505,100	31,725,835,200	45.06	98.76	10.99
**3054**	30,083,008,800	29,977,702,500	43.44	99.03	10.57
**3058**	26,370,726,900	26,185,500,900	43.64	98.85	9.58
**3068**	26,549,333,400	26,255,199,600	45.62	98.21	9.43
**3090**	28,875,465,000	28,777,767,000	45.63	98.67	10.07
**3106**	26,807,389,500	26,567,910,000	44.91	98.44	9.34
**3110**	29,028,225,300	28,807,300,800	44.43	98.54	10.09
**3134**	29,200,247,400	29,109,893,100	44.36	98.76	10.25
**3136**	27,385,819,500	27,139,808,400	45.2	98.46	9.47
**3148**	28,307,922,000	27,962,590,500	44.64	98.64	9.62
**3158**	31,579,310,100	31,484,169,000	42.99	98.97	11.26
**3160**	26,137,512,600	25,977,866,100	44.83	97.76	9.33
**3178**	26,174,787,900	25,891,073,400	45.47	98.25	9.24

**3204**	26,230,839,600	26,119,731,300	44.2	98.79	9.5
**3206**	28,884,262,500	28,647,057,900	44.86	98.15	9.75
**3212**	24,548,508,600	24,298,784,100	45.3	98.21	8.73
**4002**	26,048,211,300	25,864,923,600	45.53	98.31	9.34
**4004**	27,032,655,300	26,320,440,000	45.31	97.74	8.93
**4068**	24,994,672,800	24,834,152,100	44.14	98.59	8.79
**4500**	26,368,057,800	26,317,224,600	43.8	98.87	9.44
**4502**	26,456,618,100	26,247,536,400	44.98	98.47	9.48
**4904**	28,501,330,200	28,233,558,000	45.28	98.62	9.95
**5104**	32,556,445,200	32,284,774,200	45.09	98.57	11.62
**6002**	26,342,670,600	26,016,514,800	41.97	98.12	9.26
**6004**	25,150,688,700	24,915,823,500	45.81	98.25	8.88
**6018**	29,854,977,600	29,790,804,900	43.56	98.87	10.63
**6102**	24,169,342,500	24,038,244,900	44.29	98.67	8.48
**6104**	26,939,061,300	26,634,402,000	44.55	98.64	9.22
**6114**	27,161,812,800	26,841,708,600	45.25	98.27	9.56
**6218**	26,729,422,800	26,423,940,900	44.09	98.72	9.29
**6302**	25,494,284,700	25,323,065,700	44.2	98.9	9.06
**7006**	27,212,865,600	27,009,397,200	44.65	98.02	9.61
**7052**	27,143,605,200	26,907,215,700	45.23	98	9.32
**8002**	25,170,041,100	25,043,411,700	44.14	98.91	9.05
**8004**	25,080,276,600	24,903,587,100	45.57	97.12	8.45
**8006**	27,067,007,700	26,770,761,300	44.87	98.47	9.66
**8018**	31,997,469,300	31,793,161,200	43.33	98.81	11.1
**8054**	26,167,080,300	26,103,089,700	43.57	98.8	9.48
**8110**	27,120,856,200	26,691,318,900	44.86	98.58	9.15
**8238**	24,807,944,100	24,582,424,200	44.05	98.49	8.92
**8240**	24,415,853,700	24,223,711,500	45.63	97.72	8.77
**8300**	29,035,125,900	28,735,608,900	44.59	98.46	10.29
**8312**	26,365,672,200	26,194,489,200	45.77	98.54	9.36
**8322**	28,656,599,100	28,455,256,800	44.97	98.67	10.26
**9000**	26,741,786,400	26,487,174,000	45.28	98.75	9.28
**9004**	29,961,091,500	29,708,868,900	43.81	98.64	10.76
**9104**	29,722,211,100	29,477,367,900	44.8	98.49	10.22
**9304**	28,510,209,600	28,166,574,600	44.38	98.49	10.09
**9316**	30,153,798,300	29,834,143,800	46.41	97.95	10.61
**9404**	26,832,184,200	26,620,603,200	44.8	97.95	9.12
**9406**	27,372,590,400	27,145,541,400	45.62	98.11	9.75
**9410**	27,199,955,100	27,025,885,200	43.83	98.49	9.28
**9412**	28,078,182,900	27,888,406,200	45.47	98.3	9.75
**9418**	26,480,024,700	26,222,309,700	45.61	98.09	9.49

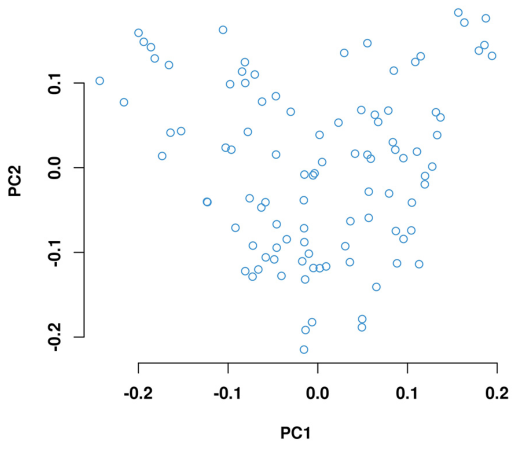

Figure S1 Principal component analysis of Qingping pigs.

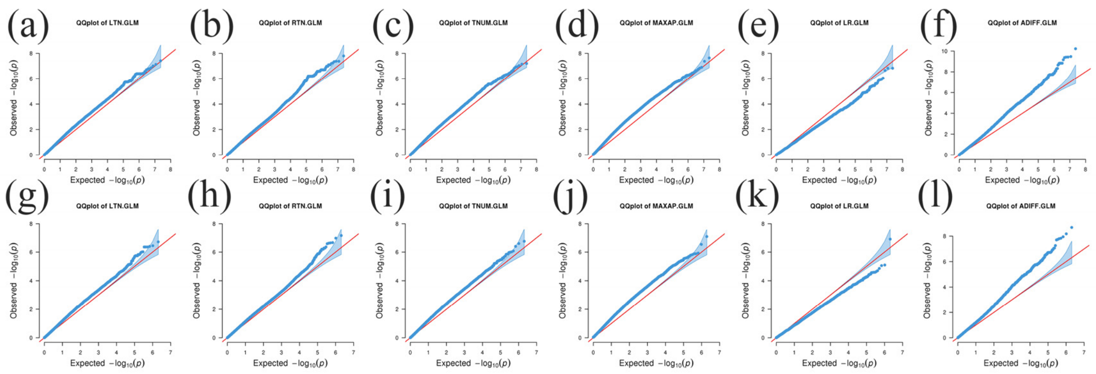

Figure S2 The Q-Q plots of the GLM GWAS for teat number-related traits in Qingping pigs, including LTN, RTN, TNUM, MAXAP, L-R and ADIFF. (a-f) The Q-Q plots of GLM GWAS based on SNPs. (g-l) The Q-Q plots of GLM GWAS based on Indels.

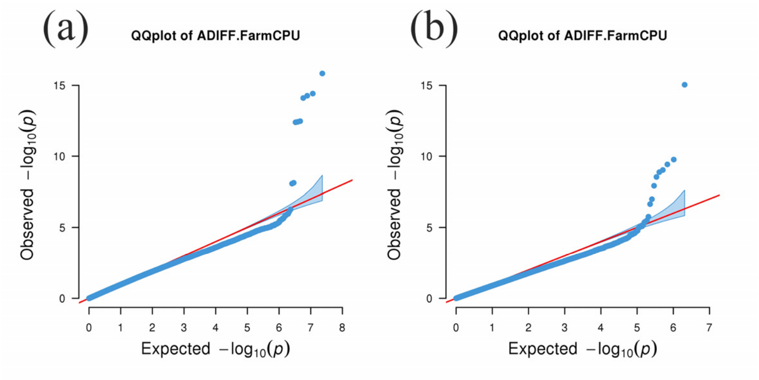

Figure S3 The Q-Q plots of the FarmCPU GWAS for ADIFF in Qingping pigs. (a) The Q-Q plot of FarmCPU GWAS based on SNPs. (b) The Q-Q plot of FarmCPU GWAS based on Indels.

Table S2 Annotated genes within 1-Mb regions of significant SNPs and Indels.

genes							
UVRAG	*SLC12A8*	*OSBPL6*	*APOD*	*KRT1*	*BCO1*	*HHAT*	*LANCL2*
WNT11	*TOP2B*	*PRKRA*	*BDH1*	*KRT77*	*PKD1L2*	*OR52N5*	*VOPP1*
GVQW3	*NGLY1*	*PJVK*	*WDR72*	*KRT3*	*GCSH*	*OR52W1*	*PGAM2*
EMSY	*BRINP3*	*FKBP7*	*U2*	*KRT4*	*C16orf46*	*OR56B4*	*DBNL*
LRRC32	*PSD3*	*PLEKHA3*	*KRTAP11-1*	*KRT79*	*ATMIN*	*OR56A1*	*UBE2D4*
TSKU	*PRNP*	*COPS8*	*TIAM1*	*KRT78*	*CENPN*	*OR56B1*	*URGCP*
ACER3	*PRND*	*ssc-mir-7141*	*SLC35F1*	*KRT8*	*CDYL2*	*WSB1*	*TBX5*
B3GNT6	*RASSF2*	*COL6A3*	*NUS1*	*KRT18*	*DYNLRB2*	*KSR1*	*RBM19*
CAPN5	*SLC23A2*	*MLPH*	*DCBLD1*	*EIF4B*	*WWOX*	*NOS2*	*LHX5*
MYO7A	*RPL10L*	*PRLH*	*ROS1*	*TNS2*	*CLEC3A*	*NLK*	*SDSL*
MMRN1	*MDGA2*	*RAB17*	*VGLL2*	*SPRYD3*	*VAT1L*	*TMEM97*	*SDS*
SNCA	*CETN3*	*RBM44*	*U2*	*SOAT2*	*NUDT7*	*IFT20*	*PLBD2*
TIGD2	*MBLAC2*	*RAMP1*	*CCDC141*	*CSAD*	*ADAMTS18*	*TNFAIP1*	*DTX1*
FAM13A	*LYSMD3*	*SCLY*	*SESTD1*	*ZNF740*	*ACSBG1*	*VTN*	*RASAL1*
SALL1	*ADGRV1*	*ESPNL*	*FBXL4*	*ITGB7*	*IDH3A*	*POLDIP2*	*CFAP73*
CYLD	*XPO6*	*FAT1*	*FAXC*	*RARG*	*CIB2*	*TMEM199*	*DDX54*
NOD2	*GSG1L*	*MTNR1A*	*PNISR*	*IRX6*	*SH2D7*	*SEBOX*	*TPCN1*
SNX20	*KATNIP*	*NPVF*	*USP45*	*OGFOD1*	*TBC1D2B*	*SARM1*	*RITA1*
NKD1	*GTF3C1*	*C7orf31*	*REV3L*	*NUDT21*	*ADAMTS7*	*SLC46A1*	*IQCD*
BRD7	*IL21R*	*CYCS*	*TRAF3IP2*	*AMFR*	*MORF4L1*	*SLC13A2*	*ZNF22*
ADCY7	*IL4R*	*OSBPL3*	*FYN*	*GNAO1*	*CTSH*	*FOXN1*	*RASSF4*
LTBP1	*NSMCE1*	*GSDME*	*CCN6*	*CES5A*	*RASGRF1*	*ZNF395*	*DEPP1*
TTC27	*KDM8*	*MPP6*	*TUBE1*	*SLC6A2*	*ANKRD34C*	*ssc-mir-124a-2*	*TMEM72*
BIRC6	*ANGEL2*	*MAP2K6*	*FAM229B*	*LPCAT2*	*TMED3*	*FZD3*	*CXCL12*
CEP120	*VASH2*	*ABCA5*	*LAMA4*	*MMP2*	*MINAR1*	*EXTL3*	*CYP4F22*
CSNK1G3	*FLVCR1*	*ABCA6*	*PTPRZ1*	*IRX5*	*ssc-mir-184*	*INTS9*	*PGLYRP2*
DMXL1	*SPATA45*	*ABCA9*	*FAM3C*	*IRX3*	*PSAPL1*	*HMBOX1*	*RASAL3*
TNFAIP8	*TATDN3*	*ABCA8*	*WNT16*	*DMP1*	*SORCS2*	*GTF3C3*	*WIZ*
HSD17B4	*NSL1*	*FAM20A*	*CPED1*	*DSPP*	*TADA2B*	*C2orf66*	*AKAP8L*
FAM170A	*BATF3*	*PRKAR1A*	*ING3*	*SPARCL1*	*BLOC1S4*	*PGAP1*	*AKAP8*
TRIML1	*FAM71A*	*WIPI1*	*LRATD1*	*HSD17B11*	*PPP2R2C*	*ANKRD44*	*BRD4*
TRIML2	*ATF3*	*ARSG*	*PDCL3*	*KLHL8*	*WFS1*	*SF3B1*	*EPHX3*
ZFP42	*PACC1*	*SLC16A6*	*CHST10*	*AFF1*	*MAN2B2*	*CCDC150*	*NOTCH3*
TMEFF2	*NENF*	*AMZ2*	*LONRF2*	*SLC10A6*	*OR56A4*	*MFAP3*	*ILVBL*
NXPH2	*PPP2R5A*	*GNA13*	*REV1*	*PTPN13*	*TRIM3*	*FAM114A2*	*SYDE1*
PHYHIPL	*DTL*	*UQCRFS1*	*COX7A2L*	*MAPK10*	*HPX*	*GRIA1*	*OR1I1*
FAM13C	*INTS7*	*VSTM2B*	*EML4*	*SEC16B*	*APBB1*	*SLC7A2*	*CASP14*
SLC16A9	*LPGAT1*	*POP4*	*PKDCC*	*RASAL2*	*SMPD1*	*PDGFRL*	*CCDC105*
CCDC6	*ARHGEF3*	*PLEKHF1*	*KRT80*	*RFX6*	*CAVIN3*	*MTUS1*	*SLC1A6*
RBM11	*IL17RD*	*C19orf12*	*KRT7*	*GPRC6A*	*CNGA4*	*FGL1*	*MTUS2*
HSPA13	*HESX1*	*URI1*	*KRT85*	*FAM162B*	*FAM160A2*	*PCM1*	*SLC7A1*
NRIP1	*APPL1*	*SLC7A11*	*KRT84*	*KPNA5*	*C11orf42*	*ASAH1*	*KATNAL1*
RUBCN	*ASB14*	*TNIK*	*KRT82*	*ZUP1*	*OR52E4*	*FRG1*	*USPL1*
FYTTD1	*DNAH12*	*PLD1*	*KRT75*	*RSPH4A*	*TRIM22*	*NPY*	*GHSR*
LRCH3	*PDE12*	*TMEM212*	*KRT5*	*PTP4A1*	*RPS6KC1*	*STK31*	*NCEH1*
IQCG	*ARF4*	*FNDC3B*	*KRT71*	*PHF3*	*NEK2*	*FAM221A*	*ECT2*
LMLN	*DENND6A*	*FAM43A*	*KRT74*	*TMEM117*	*SLC30A1*	*UPP1*	*SPATA16*
OSBPL11	*SLMAP*	*XXYLT1*	*KRT72*	*NELL2*	*RD3*	*C7orf57*	
SNX4	*RBM45*	*ACAP2*	*KRT73*	*DBX2*	*TRAF5*	*SUN3*	
ZNF148	*U1*	*U1*	*KRT2*	*GAN*	*RCOR3*	*HUS1*	
